# Probiotic Response to *Limosilactobacillus reuteri* DSM 32203 in Two Small-Breed Dogs Differing in Body Size

**DOI:** 10.3390/microorganisms14102172

**Published:** 2026-09-26

**Authors:** Benedetta Belà, Daniele Di Simone, Isa Fusaro, Alessandro Gramenzi

**Affiliations:** 1 Department of Veterinary Medicine, University of Teramo, Piano D’Accio, 64100 Teramo, Italy; 2 Department of Economics and Finance, University of Bari, Largo Abbazia Santa Scolastica 53, 70124 Bari, Italy

**Keywords:** body condition score, fecal score, intestinal microbiota, *L. reuteri*, probiotics, small-breed dogs

## Abstract

Probiotic effects in dogs may depend not only on the bacterial strain but also on host-related characteristics. Differences in body size are associated with distinct gastrointestinal and microbial features that may influence responses to nutritional interventions. This study evaluated the effects of *Limosilactobacillus reuteri* DSM 32203 in healthy small-breed dogs and investigated whether responses differed between Chihuahua and Dachshund dogs with different body weights. Healthy adult dogs were supplemented daily with *L. reuteri* DSM 32203, and body weight, body condition score, fecal score, fecal moisture, total lactobacilli, and total coliforms were evaluated throughout the study. Supplementation improved fecal quality, with increased consistency and reduced moisture, and fecal scores reaching or approaching the optimal value. An increase in total lactobacilli and a reduction in total coliforms was also observed. These findings are consistent with previous observations obtained with the same strain in other canine breeds. However, the magnitude of the reduction in total coliforms differed from that observed in our previous studies in larger breeds. Because breed, body size, and diet were not independently controlled, these cross-study differences cannot be directly attributed to body size alone. Although body size cannot be established as a direct causal factor due to potential confounding factors such as dietary differences, these findings provide preliminary evidence that host phenotypes may contribute to probiotic responsiveness.

## 1. Introduction

The domestic dog (*Canis lupus familiaris*) represents a unique model in which marked differences in body size have developed within a single species because of selective breeding. Adult body weight may range from less than 2 kg in some toy breeds to more than 50 kg in giant breeds, and this variation is accompanied by differences in gastrointestinal anatomy and physiology. Body size has been associated with changes in gastrointestinal dimensions, colonic transit, intestinal permeability, fermentative activity, and fecal water content, suggesting that the digestive environment is not uniform across canine breeds [1,2,3,4,5]. These differences are of relevance when nutritional interventions are intended to modify intestinal function, because the physiological conditions encountered by a dietary component or microorganism may vary substantially among dogs of different sizes [2,3,6]. The intestinal microbiota is an integral component of the canine gastrointestinal ecosystem, contributing to nutrient metabolism and fermentation, modulation of intestinal barrier function, and bidirectional interactions with the host [7,8,9,10]. Although the general structure and functional relevance of the canine gut microbiota are increasingly well characterized, substantial inter-individual variation has been reported, with microbial composition influenced by multiple intrinsic and extrinsic factors, including breed, age, body condition, body size, diet, and living environment [11,12,13]. Importantly, evidence also suggests that body size may contribute to differences in the canine fecal microbial ecosystem. Middleton et al. [6] identified metabolic, clinical, and microbiota differences between small and larger dogs maintained on the same diet, suggesting that some metabolic and microbial characteristics may be associated with host body size rather than being solely attributable to dietary differences. More recently, a comprehensive review of canine digestive physiology highlighted associations between body size and several gastrointestinal and fecal parameters, particularly within the colonic compartment, including intestinal transit time, permeability, fiber degradation, fecal short-chain fatty acids, and fecal water content [3]. Subsequent experimental work further demonstrated that nutritional and physicochemical conditions representative of different dog sizes can reshape canine microbiota structure and function, including microbial fermentation activity [5]. These observations are particularly relevant to the use of probiotics. Probiotics are defined as live microorganisms that, when administered in adequate amounts, confer a health benefit on the host, and their effects are increasingly investigated as a means of modulating intestinal microbial communities and gastrointestinal function [14,15,16,17]. In dogs, probiotic supplementation has been associated with changes in fecal characteristics, intestinal microbial communities, microbial metabolites, and selected host responses. However, the available evidence remains heterogeneous, and comparisons among studies are complicated by differences in probiotic strains, doses, administration protocols, diets, host characteristics, and outcome measures [18,19]. Consequently, probiotic effects should not be attributed solely to the bacterial species, as strains belonging to the same species may exhibit distinct biological properties and probiotic potential [18,20]. The strain-dependent nature of probiotic effects raises an important question that has received comparatively little attention in canine nutrition: to what extent can characteristics of the host influence the response to a defined probiotic strain? Recent evidence in healthy dogs indicates that the magnitude of the microbiota response to probiotic supplementation can vary among individuals and is associated with the microbial profile at baseline, supporting the relevance of host-related factors in probiotic responsiveness [19]. If dogs of different sizes differ in gastrointestinal anatomy, intestinal transit, water handling, fermentation, and microbial composition, the intestinal environment in which an exogenous microorganism acts may also differ according to host size [3]. It is therefore plausible that the magnitude or temporal pattern of the response to a given probiotic strain may vary across canine phenotypes. Among lactic acid bacteria investigated for canine applications, *Limosilactobacillus reuteri* has attracted interest because of its potential to interact with the intestinal microbiota and influence microbial composition and activity [18,21]. Nevertheless, the biological effects of *L. reuteri* remain strain-dependent, and effects observed with one strain should not be assumed to be transferable to other strains of the same species [18,20]. The strain *L. reuteri* DSM 32203 has previously been investigated in dogs, including the French Bulldog and Golden Retriever, and has shown potential to improve fecal consistency through a reduction in fecal moisture when administered as a feed additive [22,23,24]. The preparation containing *L. reuteri* DSM 32203 was subsequently authorized in the European Union as a zootechnical feed additive for dogs [25]. However, the response of small-breed dogs to this defined strain remains comparatively underexplored. Small-breed dogs are particularly relevant in this context because they cannot simply be considered scaled-down versions of larger dogs: body size is associated with differences in gastrointestinal anatomy and physiology, intestinal transit, fecal water content, fermentation-related parameters, and aspects of the fecal microbiota [3]. Studies comparing healthy dogs have also demonstrated that individual characteristics can be associated with measurable differences in fecal microbial communities, emphasizing the importance of considering host-related variables when interpreting microbiota-directed interventions [26]. Nevertheless, experimental studies investigating whether a defined probiotic strain produces consistent effects among dogs differing in body size remain limited. The present study was designed to investigate the effects of *L. reuteri* DSM 32203 supplementation in two small-breed populations, Chihuahua and Dachshund dogs. These breeds were selected because, despite both being classified as small dogs, they differ substantially in body weight and therefore provide an opportunity to investigate the response to the same probiotic strain in two distinct small-breed phenotypes. The effects of supplementation were evaluated longitudinally using body weight, body condition score, fecal score, fecal moisture, and selected fecal bacterial populations as outcome measures. The primary objective was to determine whether daily supplementation with *L. reuteri* DSM 32203 could improve fecal quality and modulate selected bacterial populations in healthy small-breed dogs. More broadly, the study aimed to extend previous observations obtained with the same probiotic strain in other canine breeds and to investigate whether the response to a defined probiotic strain is comparable between small-breed dogs differing in body weight. We hypothesized that *L. reuteri* DSM 32203 would improve fecal consistency and modify selected bacterial populations in both Chihuahua and Dachshund dogs, while the comparison of these two small-breed phenotypes would provide preliminary insight into whether the magnitude and pattern of the response to the same probiotic strain are broadly consistent or differ according to body weight, keeping in mind potential confounding factors such as dietary background.

## 2. Materials and Methods

### 2.1. Animals and Study Design

A total of 60 Chihuahua and Dachshund dogs (comprising 26 males and 34 non-pregnant females across both breeds, as detailed in Table 1), aged >1 year, were involved in the study and randomly assigned to the control group (CTR; *n* = 30: 15 Chihuahuas + 15 Dachshunds) and experimental group (LACTO; *n* = 30: 15 Chihuahuas + 15 Dachshunds). Animals were divided randomly (SurveyMonkey Excel) into the two groups, and the group assignment was organized following standard housing protocols within two authorized breeding establishments specialized in Chihuahuas and Dachshunds. The experimental group was supplemented with *Limosilactobacillus reuteri* DSM 32203, while the control group did not receive the probiotic and was supplemented with maltodextrin used as a placebo. The two dog breeds were analyzed individually to evaluate any differences inherent to the breed itself or to the diet administered. Table 1 reports age, sex and body weight of each dog of the two breeds belonging to the control and experimental groups. Cleaning and disinfecting procedures of the single fences were carried out and the animals were individually stabled. Moreover, the animals were evaluated daily by a veterinarian for any health and welfare concerns throughout the experimental period. The study lasted a total of 49 days, comprising a 14-day acclimatization period followed by a 35-day intervention. The conclusion of the 14-day acclimatization phase directly coincided with day 0 of the study (T0), marking the official start of the intervention period.

### 2.2. Feed Supplement and Diet

All dogs had free access to potable water and were fed with two different commercial petfood: Chihuahua dogs consumed Royal Canin mini adult extruded feed while the Dachshund dogs ate Eukanuba adult small breed food. For the entire duration of the study, feed was administered to each animal once a day based on the energy requirements of each dog (adult dogs: 110 kcal × BW^0.75^ kg) [27]; Table 2 reports the analytical components of each petfood administered. Dogs belonging to the experimental group received commercial feed with the addition of 10 g/100 kg of *Limosilactobacillus reuteri* DSM 32203, corresponding to 5 × 10^9^ colony-forming units (CFU)/kg food; the process to obtain the right probiotic amount consisted of using 50 g of the feed additive (standard concentration ≥ 1.0 × 10^11^ CFU/g), pre-mixed in the laboratory with 9950 g of maltodextrins. Then, a total of 20 g of this pre-mixture was added daily to each 980 g of commercial feed in the bowl. The control group received the commercial diet, with the addition of 20 g of maltodextrin in 980 g of dog feed (placebo).

### 2.3. Data Collection

#### 2.3.1. Body Weight and Body Condition Score Assessment

Body weight (BW) and body condition score (BCS) were recorded at the beginning of the experiment (day 0 = T0) after one week (T1), after 2 weeks (T2), after 3 weeks (T3), after 4 weeks (T4) and at the end of the study (T5) according to the American Animal Hospital Association (AAHA) Nutritional Assessment Guidelines for Dogs and Cats [28]. The body weight of each dog was measured by the same operator at the same time (morning, before feed administration) with the same instrument, while the body composition assessment was carried out by visual examination and palpation of the animal on a scale between 1 and 9 where a score of 4 or 5 reflects the ideal body condition [29].

#### 2.3.2. Fecal Scoring and Fecal Moisture Analysis

A 7-point scoring chart was used for the analysis of the fecal score according to the Nestle Purina Fecal Scoring System, while for the analysis of fecal moisture (FM), the fecal samples were collected from each dog at T0, T1, T2, T3, T4 and T5 and then stored at +4 °C until they were brought to the laboratory, where they were stored at −20 °C. 5–10 g of stool was weighed and dried in an oven at a temperature of 105–110 °C for 20–24 h, cooled down in a desiccator for another 20–24 h, after which the fecal moisture content was calculated as the lost weight after desiccation.

#### 2.3.3. Microbiological Analysis

Microbiological analysis was conducted to identify and quantify specific intestinal bacterial species. One gram of fresh stool was used to carry out this analysis at four specific time points: T0, T1, T3 and T5. Briefly, fresh stool was diluted in sterile saline solution with a ratio of 1:10 and then vortexed for two minutes to obtain a homogeneous suspension, which was plated on different culture media for total bacterial counts and identification; for *Escherichia coli* and total coliforms, eosin methylene blue agar (Oxoid, Rodano, Milan, Italy) was used, with an incubation of 24 h at 37 °C. *E. coli* colonies show growth with a green metallic reflex, while coliforms show growth with blue, red, or uncolored colonies. For the enumeration of total lactobacilli, De Man, Rogosa, and Sharpe (MRS) agar was utilized in compliance with standardized microbiological protocols and regulatory frameworks established for probiotic feed additives (e.g., EFSA guidance). While MRS agar is broadly selective for the lactic acid bacteria (LAB) group, it was implemented under controlled anaerobic incubation parameters (at 37 °C for 48 h) to specifically quantify the total lactobacilli population. All the analysis was performed in duplicate.

### 2.4. Statistical Analysis

For statistical analysis, a linear mixed model with repeated measurements was used, which allows the estimation of the parameters considering both random effect and fixed effect [30]. The model has been estimated as the following:

yi,j,k = μ + Si + Gj +Tk +Gj ∗ Tk + ei,j,k

where y = dependent variable (FM, FS, BW, BCS, LB, COLI); μ = overall mean; Si = fixed effect of the ith sex (I = 1, 2); Gj = fixed effect of the jth group (j = 1, 2); Tk = fixed effect of the kth time point (corresponding to the scheduled sampling intervals T0–T5 for clinical and physical parameters, and T0, T1, T3, T5 for microbiological analyses); and ei,j,k = residual error term. To properly account for the longitudinal structure and repeated observations within individual subjects over time, an autoregressive covariance structure AR(1) was incorporated into the mixed model. Sex and breed-specific differences were fully evaluated within the statistical framework, and results were presented separately for each breed to properly illustrate phenotypic responses. The software used was R Core Team (version 4.0.0, 2020), R being a language and environment for statistical computing [31], and for the different analysis, the mixed model [32] and the least squares [33] methods were used. Least squares means were estimated, and they have been statistically tested using Student’s *t* test (with Tukey *p*-value adjustment). To be able to describe the goodness of fit of the mixed model, we used the R squared described by Nakagawa et al. [34]. No outliers and missing data were found.

## 3. Results

### 3.1. Body Weight and Body Condition Score

The results of the study show how *L. reuteri* DSM 32203 supplementation has no influence on the weight (Table 3) and body condition (Table 4) of Chihuahua and Dachshund adult healthy dogs; in fact, the body weight of each dog remained almost constant throughout the entire duration of the study.

### 3.2. Fecal Moisture

Fecal moisture (Table 5) decreased in the group of dogs treated with the probiotic *L. reuteri* DSM 32203 compared to the control one; similar results were reported in both Chihuahua and Dachshund dogs. At the end of the 35 days of the study, Chihuahuas supplemented with *L. reuteri* DSM 32203 showed a decrease in fecal moisture of 0.20 (g/g) compared to T0 (beginning of the study), while the Dachshund dogs showed a decrease of 0.24 (g/g) (Table 5A,B).

### 3.3. Fecal Score

Furthermore, fecal scores improved significantly in probiotic-supplemented dogs (Table 6). Starting from the second week of study (T2), the Chihuahua group treated with *L. reuteri* DSM 32203 reported a fecal score value of 2.09 (Table 6A), very close to the value considered optimal, i.e., 2. The Dachshund dogs reported the same excellent score starting from the fourth week of study (T4), while in the respective control group no similar improvement was observed (Table 6B).

### 3.4. Microbiological Analyses

Regarding the microbiological analysis, the trend of lactobacilli is very similar between Chihuahua and Dachshund dogs supplemented with *L. reuteri* DSM 32203; in both groups a clear increase in this bacterial genus is observed starting from the third week of the study, continuing to increase until the end of the experiment (Table 7). Specifically, in the group of Chihuahuas treated with *L. reuteri* DSM 32203, a significant increase in lactobacilli was recorded, rising from an initial concentration of 4.72 ± 0.06 log CFU/g to 5.63 ± 0.06 log CFU/g after 35 days of study (Table 7A). As for the Dachshunds, the LACTO group went from an initial concentration of 4.80 ± 0.06 log CFU/g to reach a concentration of 5.62 ± 0.06 log CFU/g at the end of the experiment (Table 7B).

In addition, total coliforms decreased in the probiotic-treated groups compared to the control groups. Specifically, the LACTO group in Chihuahua dogs reported an initial concentration of 4.65 ± 0.08 log CFU/g and a final concentration of 3.94 ± 0.08 log CFU/g, while the control group maintained an initial concentration of 4.73 ± 0.08 log CFU/g and a final concentration of 4.71 ± 0.08 log CFU/g (Table 8A). A similar decrease was observed in Dachshunds: the LACTO group showed an initial coliform concentration of 4.55 ± 0.08 log CFU/g, which dropped to 3.96 ± 0.08 log CFU/g at the end of the trial, whereas the control group showed an initial count of 4.60 ± 0.08 log CFU/g and a final count of 4.68 ± 0.08 log CFU/g (Table 8B).

## 4. Discussion

### 4.1. Body Weight and Body Condition Score

The potential beneficial effects of probiotic supplementation in dogs have been widely investigated; however, the biological effects of probiotics are highly dependent on the specific strain used. Strains belonging to the same bacterial species may differ substantially in their functional properties and in their interactions with the host, supporting the concept of strain-specificity in probiotic activity [18,19,20]. Therefore, results obtained with one probiotic strain cannot necessarily be extrapolated to other strains, even when they belong to the same bacterial species. In dogs, relatively few studies have investigated the effects of *Limosilactobacillus reuteri*, and limited information is available on whether the response to a defined probiotic strain may vary according to host characteristics such as body size. This aspect is of particular interest in the domestic dog, which represents a unique species characterized by marked differences in body weight and morphology among breeds. As previously described, dogs may range from very small breeds such as Chihuahuas to giant breeds such as Saint Bernards [35]. Such differences are not limited to body weight but are also associated with relevant variations in gastrointestinal anatomy and physiology. Digestion in dogs is a complex process involving physicochemical, mechanical, and microbial mechanisms, and the gastrointestinal environment may differ substantially according to body size. The increasing recognition of the role of the intestinal microbiota in canine health has led to the widespread use of nutritional strategies aimed at modulating the intestinal microbial ecosystem, including probiotics, prebiotics, postbiotics, and other nutraceutical products. However, the potential effects of these interventions should be considered in relation to the physiological characteristics of the host. This concept is also reflected by the development of commercial diets formulated according to specific breed characteristics, body size, or predispositions [36,37,38,39]. Despite the relevance of these differences, the influence of body size on canine digestive physiology remains relatively poorly investigated. Deschamps et al. [3], in a comprehensive review of the available literature, identified a limited number of studies addressing digestive differences among dogs of different sizes, with a substantial proportion of the available evidence deriving from individual breeds rather than direct comparisons among size categories. This limited evidence makes it difficult to establish a comprehensive picture of how body size affects the different phases of canine digestion. The available data suggest that the relationship between body size and gastrointestinal motility is not uniform across the gastrointestinal tract. Studies evaluating gastric emptying and small intestinal transit have not consistently demonstrated a clear association with body weight, suggesting that these parameters may be influenced more strongly by factors such as food characteristics [40,41,42,43]. In contrast, differences in large intestinal physiology appear to be more closely related to body size. Hernot et al. [1] reported a longer large intestinal transit time in larger dogs and identified a correlation between transit time and body size. In addition, the length, area, and volume of the large intestine increase with body size, potentially contributing to differences in water handling, fermentation, and fecal characteristics. These physiological differences may also influence the intestinal microbial ecosystem. Although the overall functions performed by the intestinal microbiota appear to be broadly conserved among dogs, microbial composition can vary considerably between individuals and may be influenced by host-related factors, including body size [44]. Fecal concentrations of short-chain fatty acids have been reported to increase with increasing body weight [45], potentially reflecting differences in colonic fermentation. Longer colonic transit may provide microorganisms with more time for substrate utilization and fermentation, thereby influencing microbial metabolites and fecal characteristics. At the same time, excessive fermentation and the consequent accumulation of osmotically active metabolites may contribute to alterations in fecal water content. Increased intestinal permeability has also been described in large-breed dogs and may further contribute to differences in intestinal water and electrolyte handling [2]. Taken together, these observations indicate that dogs of different sizes may provide distinct gastrointestinal environments for the activity of dietary microorganisms. It is therefore reasonable to hypothesize that the response to the same probiotic strain may not necessarily be identical across canine phenotypes. This consideration provided the rationale for the present study, in which the effects of *L. reuteri* DSM 32203 were evaluated in two small-breed populations differing in body weight, namely Chihuahuas and Dachshunds.

### 4.2. Fecal Moisture and Fecal Score

In the present study, supplementation with *L. reuteri* DSM 32203 was associated with an improvement in fecal quality in both breeds, as indicated by increased fecal consistency and reduced fecal moisture, with fecal scores reaching or approaching a score of 2, considered indicative of optimal fecal quality. These findings are consistent with previous evidence regarding the ability of this strain to improve fecal consistency in healthy dogs. The improvement in fecal parameters may reflect a more favorable intestinal environment and improved fecal water handling, although the present study does not allow direct conclusions to be drawn regarding intestinal nutrient absorption.

### 4.3. Microbiological Analyses

The administration of the probiotic was associated with an increase in total lactobacilli and a reduction in total coliforms. The increase in lactobacilli is consistent with the administration of a viable lactic acid bacterium and with the results previously obtained with the same strain in other canine breeds. In our previous studies conducted in Golden Retrievers and French Bulldogs, supplementation with *L. reuteri* DSM 32203 similarly resulted in an increase in total lactobacilli [22,23]. The reduction in total coliforms observed in the present study is also of interest, although coliform counts should be interpreted as an indicator of changes in this bacterial group rather than as a direct measure of pathogenic bacteria. When the present findings are considered together with our previous observations, the response of total coliforms appears to differ according to the canine population investigated. Descriptively, the reduction in total coliforms observed in the present study was greater in magnitude than that reported in our previous studies in Golden Retrievers and French Bulldogs [22,23]. However, these studies differed in breed, diet, and experimental conditions, and no formal statistical comparison across studies was performed. Although these observations cannot establish a direct effect of body size, they raise the possibility that the magnitude of the response to *L. reuteri* DSM 32203 may vary among dogs differing in body size. One possible explanation is that differences in gastrointestinal physiology, intestinal transit, water handling, and the composition of the resident microbiota may modify the intestinal environment in which the probiotic strain interacts with the host. However, this interpretation should be considered preliminary.

### 4.4. Limitations, Breed/Diet Confounders

The present study compared two small-breed populations and was not specifically designed to isolate the effect of body size from other breed-related characteristics. Breed-associated differences in diet, baseline microbiota, gastrointestinal physiology, and other host factors may have also contributed to the observed responses. Therefore, the greater reduction in coliforms observed in the present study should not be interpreted as evidence that *L. reuteri* DSM 32203 is intrinsically more effective in small dogs. An additional limitation is that breed and diet were not independently controlled, as Chihuahuas and Dachshunds received different commercial diets; any differences observed between breeds cannot be attributed specifically to body size, since dietary composition may also influence fecal characteristics and the intestinal microbiota. Rather, these findings provide preliminary evidence that the response to a defined probiotic strain may vary among canine phenotypes and support the need for further studies including dogs belonging to different and well-defined body-size categories under standardized dietary regimens. Overall, the present findings confirm the ability of *L. reuteri* DSM 32203 to positively influence selected fecal parameters and bacterial populations in healthy small-breed dogs. More importantly, when considered together with previous results obtained in other breeds, they suggest that host-related characteristics, including body size, may contribute to the magnitude of the response to a probiotic strain. Further controlled studies involving a broader range of canine body sizes will be necessary to determine whether the differences observed among breeds reflect a true size-related effect or a combination of body size and other breed-specific characteristics.

## 5. Conclusions

As previously mentioned, this study is the last in a series of three experiments [22,23] performed to evaluate the efficacy of the probiotic *Limosilactobacillus reuteri* DSM 32203 on body condition, fecal parameters and intestinal microbiota of healthy adult dogs of different sizes. Supplementation with *L. reuteri* DSM 32203 was associated with improved fecal parameters and changes in selected bacterial populations in healthy Chihuahua and Dachshund dogs. The observed responses support the potential efficacy of this strain in small-breed dogs. However, because breed, body size, and diet were not independently controlled, the present findings do not allow a specific effect of body size to be established. Further studies using standardized diets and a broader range of breeds and body-size categories are warranted to determine the contribution of host-related characteristics to probiotic responsiveness.

## Figures and Tables

**Table 1:** Age (month), sex, body weight (Kg) and breed of each dog enrolled in the study for both the LACTO and Control (CTR) groups.

Subject ID	Group	Age (Months)	Sex	Body Weight (kg)	Breed
1	CTR	14.8	M	2.60	Chihuahua
2	CTR	23.6	M	2.70	Chihuahua
3	CTR	23.6	M	2.50	Chihuahua
4	CTR	30.4	M	2.80	Chihuahua
5	CTR	14.8	M	2.90	Chihuahua
6	CTR	14.8	M	2.60	Chihuahua
7	CTR	30.4	F	1.80	Chihuahua
8	CTR	30.4	F	1.90	Chihuahua
9	CTR	23.6	F	1.70	Chihuahua
10	CTR	23.6	F	1.60	Chihuahua
11	CTR	14.8	F	1.80	Chihuahua
12	CTR	14.8	F	1.80	Chihuahua
13	CTR	23.6	F	1.80	Chihuahua
14	CTR	30.4	F	1.70	Chihuahua
15	CTR	30.4	F	1.60	Chihuahua
16	CTR	26.2	M	8.40	Dachshund
17	CTR	27.4	M	8.60	Dachshund
18	CTR	26.8	M	8.40	Dachshund
19	CTR	28.9	M	8.80	Dachshund
20	CTR	30.2	M	8.70	Dachshund
21	CTR	26.9	M	8.80	Dachshund
22	CTR	34.5	M	8.60	Dachshund
23	CTR	36.2	F	7.50	Dachshund
24	CTR	34.9	F	7.60	Dachshund
25	CTR	33.5	F	7.70	Dachshund
26	CTR	34.7	F	7.50	Dachshund
27	CTR	32.8	F	7.60	Dachshund
28	CTR	32.2	F	7.80	Dachshund
29	CTR	29.9	F	7.90	Dachshund
30	CTR	30.3	F	7.50	Dachshund
31	LACTO	30.4	M	2.90	Chihuahua
32	LACTO	30.4	M	2.80	Chihuahua
33	LACTO	23.6	M	2.70	Chihuahua
34	LACTO	14.8	M	2.90	Chihuahua
35	LACTO	14.8	M	2.90	Chihuahua
36	LACTO	23.6	M	2.80	Chihuahua
37	LACTO	23.6	F	1.70	Chihuahua
38	LACTO	23.6	F	1.70	Chihuahua
39	LACTO	23.6	F	1.60	Chihuahua
40	LACTO	14.8	F	1.80	Chihuahua
41	LACTO	14.8	F	1.90	Chihuahua
42	LACTO	30.4	F	1.80	Chihuahua
43	LACTO	30.4	F	1.70	Chihuahua
44	LACTO	30.4	F	1.70	Chihuahua
45	LACTO	14.8	F	1.80	Chihuahua
46	LACTO	28.8	M	8.80	Dachshund
47	LACTO	26.6	M	8.90	Dachshund
48	LACTO	25.4	M	8.70	Dachshund
49	LACTO	24.5	M	8.70	Dachshund
50	LACTO	29.7	M	8.60	Dachshund
51	LACTO	27.8	M	8.50	Dachshund
52	LACTO	30.5	M	8.80	Dachshund
53	LACTO	33.2	F	7.50	Dachshund
54	LACTO	35.6	F	7.50	Dachshund
55	LACTO	34.2	F	7.60	Dachshund
56	LACTO	31.7	F	7.70	Dachshund
57	LACTO	34.5	F	7.80	Dachshund
58	LACTO	34.2	F	7.70	Dachshund
59	LACTO	33.3	F	7.60	Dachshund
60	LACTO	31.5	F	7.90	Dachshund

**Table 2:** Analytical components of Royal Canin mini adult extruded feed (**A**) and Eukanuba adult small breed food (**B**). (**A**) Royal Canin mini adult extruded feed. (**B**) Eukanuba adult small breed food.

Parameter	Value
(**A**)	
Crude protein (%)	27.00
Crude fats (%)	16.00
Raw fiber (%)	1.30
Raw ash (%)	6.80
Metabolizable energy (kcal/kg)	3920
(**B**)	
Crude protein (%)	30.00
Crude fats (%)	18.00
Raw fiber (%)	2.00
Raw ash (%)	6.70
Metabolizable energy (kcal/kg)	3923

(**A**) Composition: dehydrated poultry protein, wheat flour, maize flour, animal fats, maize, wheat gluten, barley, maize gluten, wheat, hydrolyzed animal proteins, minerals, beet pulp, soya oil, yeast products, fructooligosaccharides, fish oil, algae oil. Nutritional additives: Vitamin A: 15700 IU, Vitamin D3: 1000 IU, Iron (3b103): 36 mg, Iodine (3b201, 3b202): 3.6 mg, Copper (3b405, 3b406): 11 mg, Manganese (3b502, 3b504): 47 mg, Zinc (3b603, 3b605, 3b606): 133 mg, Selenium (3b801, 3b811, 3b812): 0.09 mg Preservatives–Antioxidants. (**B**) Composition: dehydrated chicken and turkey (28% of which is 17% chicken, a natural source of glucosamine, chondroitin sulphate and taurine), fresh chicken (15%), corn, wheat, poultry fat, oats, barley, millet, beet pulp (3.5%), chicken gravy, fish meal (natural source of Omega 3 fatty acids), minerals (including sodium hexametaphosphate 0.35%), dehydrated whole eggs, fructo-oligosaccharides (0.38%, natural prebiotics). Nutritional additives: Vitamin A (47,749 IU/kg), Vitamin C (60 mg/kg), Vitamin D3 (1584 IU/kg), Vitamin E (265 mg/kg), Beta-Carotene (5.2 mg/kg), L-Carnitine (50 mg/kg), Copper (9 mg/kg), Iodine (1.1 mg/kg), Manganese (4 mg/kg), Zinc (100 mg/kg), Selenium (0.01 mg/kg). Antioxidants: tocopherol-rich vegetable oil extracts (86 mg/kg); flavorings: organic rosemary extract (46 mg/kg), tea extract (23 mg/kg).

**Table 3:** Effect of *Limosilactobacillus reuteri* DSM 32203 supplementation on body weight of Chihuahua (**A**) and Dachshund (**B**) adult healthy dogs. (**A**) Effect of *Limosilactobacillus reuteri* on body weight (LS Mean ± SE) of Chihuahua dogs. (**B**) Effect of *Limosilactobacillus reuteri* on body weight (LS Mean ± SE) of Dachshund dogs.

Time	CTR Group	LACTO Group	*p*-Value
(**A**)			
Overall	2.20 ± 0.05	2.23 ± 0.05	0.306
T0	2.22 ± 0.08	2.28 ± 0.08	0.850
T1	2.21 ± 0.08	2.21 ± 0.08	1.000
T2	2.21 ± 0.08	2.22 ± 0.08	1.000
T3	2.17 ± 0.08	2.23 ± 0.08	0.850
T4	2.20 ± 0.08	2.19 ± 0.08	1.000
T5	2.19 ± 0.08	2.27 ± 0.08	0.510
(**B**)			
Overall	8.08 ± 0.06	8.13 ± 0.06	0.160
T0	8.13 ± 0.10	8.19 ± 0.10	0.960
T1	8.09 ± 0.10	8.13 ± 0.10	0.990
T2	8.10 ± 0.10	8.08 ± 0.10	1.000
T3	8.08 ± 0.10	8.10 ± 0.10	1.000
T4	8.01 ± 0.10	8.11 ± 0.10	0.612
T5	8.05 ± 0.10	8.17 ± 0.10	0.340

**Table 4:** Effect of *Limosilactobacillus reuteri* DSM 32203 supplementation on body condition score of Chihuahua (**A**) and Dachshund (**B**) adult healthy dogs. (**A**) Effect of *Limosilactobacillus reuteri* on body condition score (LS Mean ± SE) of Chihuahua dogs. (**B**) Effect of *Limosilactobacillus reuteri* on body condition score (LS Mean ± SE) of Dachshund dogs.

Time	CTR Group	LACTO Group	*p*-Value
(**A**)			
Overall	4.73 ± 0.10	4.68 ± 0.10	0.463
T0	4.71 ± 0.24	4.65 ± 0.24	1.000
T1	4.75 ± 0.24	4.71 ± 0.24	1.000
T2	4.71 ± 0.24	4.71 ± 0.24	1.000
T3	4.78 ± 0.24	4.68 ± 0.24	0.990
T4	4.71 ± 0.24	4.68 ± 0.24	1.000
T5	4.71 ± 0.24	4.68 ± 0.24	1.000
(**B**)			
Overall	4.68 ± 0.10	4.72 ± 0.10	0.660
T0	4.64 ± 0.24	4.70 ± 0.24	1.000
T1	4.70 ± 0.24	4.74 ± 0.24	1.000
T2	4.70 ± 0.24	4.70 ± 0.24	1.000
T3	4.67 ± 0.24	4.77 ± 0.24	1.000
T4	4.67 ± 0.24	4.70 ± 0.24	1.000
T5	4.67 ± 0.24	4.70 ± 0.24	1.000

**Table 5:** Effect of *Limosilactobacillus reuteri* DSM 32203 supplementation on fecal moisture (expressed as g/g) of Chihuahua (**A**) and Dachshund (**B**) adult healthy dogs: results of mixed models showing least squares means ± SE in the CTR (control group) and LACTO (experimental group) dogs, for the six individual sampling times and overall, throughout the study. (**A**) Effect of *Limosilactobacillus reuteri* on fecal moisture (LS Mean ± SE) of Chihuahua dogs. (**B**) Effect of *Limosilactobacillus reuteri* on fecal moisture (LS Mean ± SE) of Dachshund dogs.

Time	CTR Group	LACTO Group	*p*-Value
(**A**)			
Overall	0.68 ± 0.01	0.60 ± 0.01	<0.001
T0	0.68 ± 0.02	0.67 ± 0.02	0.950
T1	0.71 ± 0.02	0.69 ± 0.02	0.410
T2	0.68 ± 0.02	0.63 ± 0.02	<0.001
T3	0.68 ± 0.02	0.58 ± 0.02	<0.001
T4	0.67 ± 0.02	0.52 ± 0.02	<0.001
T5	0.69 ± 0.02	0.48 ± 0.02	<0.001
(**B**)			
Overall	0.68 ± 0.01	0.57 ± 0.01	<0.001
T0	0.68 ± 0.02	0.67 ± 0.02	0.960
T1	0.68 ± 0.02	0.64 ± 0.02	0.001
T2	0.67 ± 0.02	0.60 ± 0.02	<0.001
T3	0.67 ± 0.02	0.56 ± 0.02	<0.001
T4	0.68 ± 0.02	0.50 ± 0.02	<0.001
T5	0.69 ± 0.02	0.43 ± 0.02	<0.001

**Table 6:** Effect of *Limosilactobacillus reuteri* DSM 32203 supplementation on the fecal score of Chihuahua (**A**) and Dachshund (**B**) adult healthy dogs: results of mixed models showing least squares means ± SE in CTR (control group) and LACTO (experimental group) dogs, for the six individual sampling times and overall, throughout the study. (**A**) Effect of *Limosilactobacillus reuteri* on fecal score (LS Mean ± SE) of Chihuahua dogs. (**B**) Effect of *Limosilactobacillus reuteri* on fecal score (LS Mean ± SE) of Dachshund dogs.

Time	CTR Group	LACTO Group	*p*-Value
(**A**)			
Overall	2.55 ± 0.08	2.07 ± 0.08	<0.001
T0	2.56 ± 0.18	2.66 ± 0.18	0.980
T1	2.56 ± 0.18	2.26 ± 0.18	0.037
T2	2.49 ± 0.18	2.09 ± 0.18	0.001
T3	2.62 ± 0.18	1.99 ± 0.18	<0.001
T4	2.46 ± 0.18	1.79 ± 0.18	<0.001
T5	2.62 ± 0.18	1.62 ± 0.18	<0.001
(**B**)			
Overall	2.91 ± 0.15	2.31 ± 0.15	<0.001
T0	2.93 ± 0.25	2.96 ± 0.25	1.000
T1	2.86 ± 0.25	2.56 ± 0.25	0.300
T2	3.00 ± 0.25	2.23 ± 0.25	<0.001
T3	2.86 ± 0.25	2.10 ± 0.25	<0.001
T4	2.86 ± 0.25	2.00 ± 0.25	<0.001
T5	2.93 ± 0.25	2.00 ± 0.25	<0.001

**Table 7:** Effect of *Limosilactobacillus reuteri* DSM 32203 supplementation, expressed as log CFU/g, on the total amount of Lactobacilli present in the intestinal microflora of Chihuahua (**A**) and Dachshund (**B**) adult healthy dogs: results of mixed models showing least squares means ± SE in CTR (control group) and LACTO (experimental group) dogs, for the four individual sampling times and overall, throughout the study. (**A**) Effect of *Limosilactobacillus reuteri* on total amount of Lactobacilli (LS Mean ± SE) in Chihuahua dogs. (**B**) Effect of *Limosilactobacillus reuteri* on total amount of Lactobacilli (LS Mean ± SE) in Dachshund dogs.

Time	CTR Group	LACTO Group	*p*-Value
(**A**)			
Overall	4.70 ± 0.04	5.11 ± 0.04	<0.001
T0	4.67 ± 0.06	4.72 ± 0.06	0.780
T1	4.71 ± 0.06	4.89 ± 0.06	<0.001
T3	4.70 ± 0.06	5.22 ± 0.06	<0.001
T5	4.71 ± 0.06	5.63 ± 0.06	<0.001
(**B**)			
Overall	4.72 ± 0.04	5.15 ± 0.04	<0.001
T0	4.71 ± 0.06	4.80 ± 0.06	0.102
T1	4.73 ± 0.06	4.96 ± 0.06	<0.001
T3	4.72 ± 0.06	5.24 ± 0.06	<0.001
T5	4.72 ± 0.06	5.62 ± 0.06	<0.001

**Table 8:** Effect of *Limosilactobacillus reuteri* DSM 32203 supplementation, expressed as log CFU/g, on the amount of coliforms present in the intestinal microflora of Chihuahua (**A**) and Dachshund (**B**) adult healthy dogs: results of mixed models showing least squares means ± SE in CTR (control group) and LACTO (experimental group) dogs, for the four individual sampling times and overall, throughout the study. (**A**) Effect of *Limosilactobacillus reuteri* on total amount of coliforms (LS Mean ± SE) in Chihuahua dogs. (**B**) Effect of *Limosilactobacillus reuteri* on total amount of coliforms (LS Mean ± SE) in Dachshund dogs.

Time	CTR Group	LACTO Group	*p*-Value
(**A**)			
Overall	4.70 ± 0.04	4.31 ± 0.04	<0.001
T0	4.73 ± 0.08	4.65 ± 0.08	0.514
T1	4.66 ± 0.08	4.45 ± 0.08	<0.001
T3	4.70 ± 0.08	4.21 ± 0.08	<0.001
T5	4.71 ± 0.08	3.94 ± 0.08	<0.001
(**B**)			
Overall	4.64 ± 0.04	4.28 ± 0.05	<0.001
T0	4.60 ± 0.08	4.55 ± 0.08	0.912
T1	4.57 ± 0.08	4.40 ± 0.08	0.004
T3	4.72 ± 0.08	4.23 ± 0.08	<0.001
T5	4.68 ± 0.08	3.96 ± 0.08	<0.001

## Data Availability

The original contributions presented in this study are included in the article. Further inquiries can be directed to the corresponding author.
